# Nicotine promotes atherosclerosis via ROS-NLRP3-mediated endothelial cell pyroptosis

**DOI:** 10.1038/s41419-017-0257-3

**Published:** 2018-02-07

**Authors:** Xianxian Wu, Haiying Zhang, Wei Qi, Ying Zhang, Jiamin Li, Zhange Li, Yuan Lin, Xue Bai, Xin Liu, Xiaohui Chen, Huan Yang, Chaoqian Xu, Yong Zhang, Baofeng Yang

**Affiliations:** 1 Department of Pharmacology (the State-Province Key Laboratories of Biomedicine-Pharmaceutics of China, Key Laboratory of Cardiovascular Research, Ministry of Education), College of Pharmacy, Harbin Medical University, Harbin, 150081 China; 2 Institute of Laboratory Animal Science, Chinese Academy of Medical Sciences (CAMS) and Comparative Medicine Centre, Peking Union Medical Collage (PUMC), Beijing, China; 3 Department of Inorganic Chemistry and Physical Chemistry, College of Pharmacy, Harbin Medical University, Harbin, 150081 China; 4 Institute of Metabolic Disease, Heilongjiang Academy of Medical Science, Harbin, 150086 China; 5 Department of Pharmacology and Therapeutics, Melbourne School of Biomedical Sciences, Faculty of Medicine, Dentistry and Health Sciences, The University of Melbourne, Melbourne, 3010 Australia

## Abstract

Cigarette smoking is a major risk factor for atherosclerosis and other cardiovascular diseases. Increasing evidence has demonstrated that nicotine impairs the cardiovascular system by targeting vascular endothelial cells, but the underlying mechanisms remain obscure. It is known that cell death and inflammation are crucial processes leading to atherosclerosis. We proposed that pyroptosis may be implicated in nicotine-induced atherosclerosis and therefore conducted the present study. We found that nicotine resulted in larger atherosclerotic plaques and secretion of inflammatory cytokines in ApoE^−/−^ mice fed with a high-fat diet (HFD). Treatment of human aortic endothelial cells (HAECs) with nicotine resulted in NLRP3-ASC inflammasome activation and pyroptosis, as evidenced by cleavage of caspase-1, production of downstream interleukin (IL)-1β and IL-18, and elevation of LDH activity and increase of propidium iodide (PI) positive cells, which were all inhibited by caspase-1 inhibitor. Moreover, silencing NLRP3 or ASC by small interfering RNA efficiently suppressed nicotine-induced caspase-1 cleavage, IL-18 and IL-1β production, and pyroptosis in HAECs. Further experiments revealed that the nicotine-NLRP3-ASC-pyroptosis pathway was activated by reactive oxygen species (ROS), since ROS scavenger (N-acetyl-cysteine, NAC) prevented endothelial cell pyroptosis. We conclude that pyroptosis is likely a cellular mechanism for the pro-atherosclerotic property of nicotine and stimulation of ROS to activate NLRP3 inflammasome is a signaling mechanism for nicotine-induced pyroptosis.

## Introduction

Overwhelming evidence suggests that cigarette smoking is related to several pathologic conditions, including malignancies and cardiopulmonary diseases^1, 2^. Cigarette smoking is a major preventable risk factor for atherosclerosis and cardiovascular diseases^3^, through accelerating atherosclerosis in predisposing sites, including aorta, coronary arteries, carotid and cerebral arteries, and the large arteries in the peripheral circulation^1^. More than 4000 chemical constituents can be found in cigarette smoke, of which nicotine is the principal addictive component^4^. Substantial evidence supports the promoting effect of nicotine on atherosclerosis in a long-term basis^5^, even though short term exposure to nicotine is considered relatively harmless. However, the underlying mechanisms remain largely unknown.

Atherosclerosis is a chronic inflammatory disease. Cell death and inflammation are the two critical pathological mechanisms for atherosclerosis^6, 7^. Increased number of cell death can be observed in human atherosclerotic lesions, especially in advanced plaques. Typically, cell death is primarily ascribed to apoptosis and necrosis; however, a couple of other forms of cell death have also been identified, including pyroptosis^8^. Pyroptosis is a unique form of inflammatory cell death that is mediated by inflammasome and is dependent on caspase-1 activation. Activation of caspase-1 is responsible for the maturation of pro-IL-1β and pro-IL-18^9^. Both infectious and non-infectious stimuli could trigger pyroptotic cell death. Recently, it has been reported that pyroptosis is involved in the ox-LDL-induced human macrophages death, suggesting a critical role of pyroptosis in atherosclerosis^10^.

Located at the interface between blood and interstitial tissues, endothelium constitutes a protective barrier against endogenous danger signals^11^. Endothelial cell (EC) death is a crucial and initial stage for the development of atheroseclerosis^12, 13^. Previous reports showed that caspase-1 activation in ECs can promote endothelial activation, monocyte recruitment, and atherogenesis^14^. Additionally, caspase-1 deficiency decreases atherosclerosis in apolipoprotein E-null mice^15^. Meanwhile, evidence shows that chronic nicotine exposure augments atherosclerosis by enhancing the production of pro-inflammatory cytokines, including IL-1β and TNF-α. It is therefore conceivable that ECs likely undergo a death pathway associated with inflammation^16^. Nevertheless, whether pyroptosis is involved in EC death upon nicotine exposure, and how it is related to the observed overproduction of inflammatory cytokines remain to be clarified.

Here, we present our novel findings that nicotine showed proatherogenic effects in ApoE^−/−^ mice, which was partially mediated by the pyroptosis of endothelial cells. Activation of NLRP3 inflammasome has been identified in endothelial cells when exposed to nicotine. Silencing of NLRP3 inhibited the pyroptotic response induced by nicotine. Given the important role of ROS in activating inflammasome, we also detected the role of oxidative stress in endothelial cells pyroptosis.

## Results

### Nicotine exposure promoted atherosclerotic lesions in ApoE^−/−^ mice

**Fig. 1:**
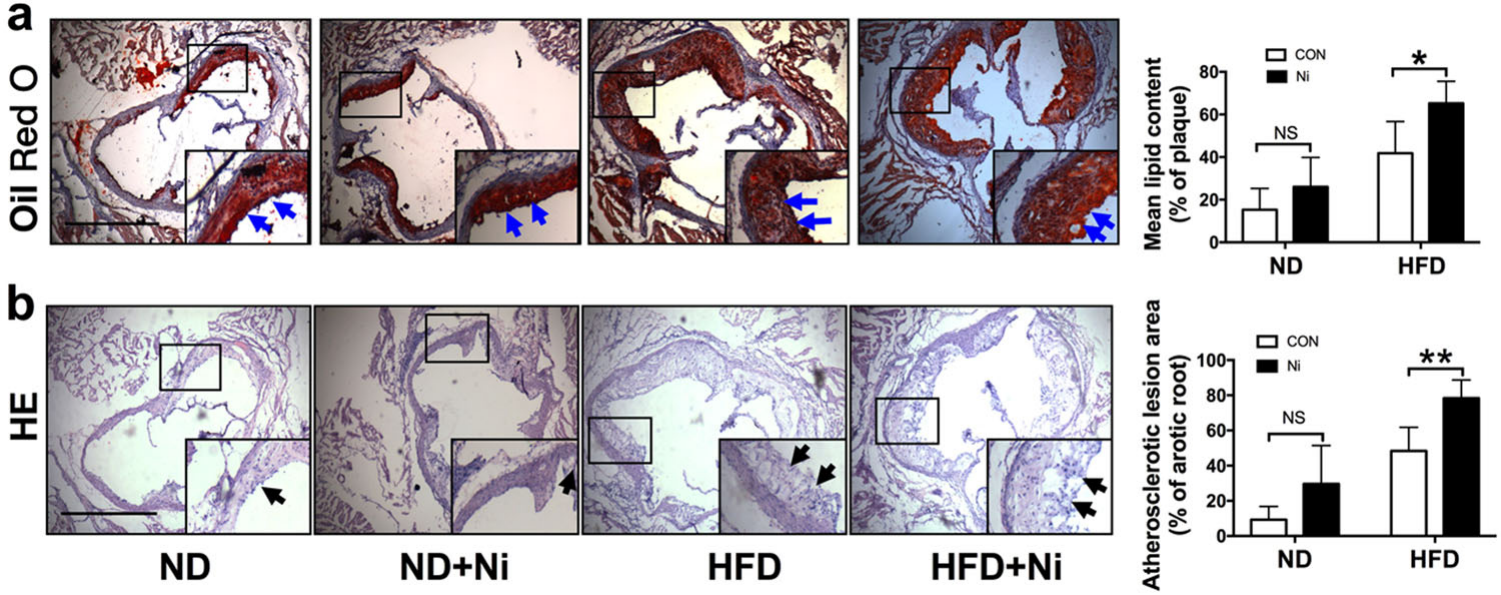
Nicotine exposure promotes atherosclerotic lesions in ApoE^−/−^ mice. **a** Representative images showing the increases of the lipid deposition by nicotine in ApoE^−/−^ mice fed with HFD (high-fat diet), but not ND (normal diet), as revealed by Oil Red O staining of aortic root sections. The right panel shows the averaged data measured from the images as shown in the left panel. Magnification: ×40. Scale bar = 2000 μm. *n* = 6 mice in each group. **b** Representative images showing the increases of the atherosclerotic lesions by nicotine in ApoE^−/−^ mice fed with HFD, as revealed by HE staining of aortic root sections. *n* = 6 mice in each group. The data are presented as the mean ± SEM. *P < 0.05, **P < 0.01

Previous study has demonstrated that nicotine induces cardiovascular diseases^17^. To dissect the role of nicotine during the progression of atherosclerosis, we performed HE and Oil Red O staining in histological sections of the aortic sinus of the ApoE^−/−^ mice. Twenty-four ApoE^−/−^ mice were divided into normal diet group (ND), high-fat diet group (HFD), ND plus nicotine (Ni) group, and HFD plus Ni group. Consistent with previous study^18^, our results showed that 12 weeks of nicotine treatment stimulated plaque formation in ApoE^−/−^ mice fed with HFD (Fig. 1). By comparison, nicotine treatment had a smaller effect on lesion areas in ApoE^−/−^ mice fed with ND.

### Endothelial cells displayed characteristic features of pyroptosis in the aorta of nicotine-induced ApoE^−/−^ mice

**Fig. 2:**
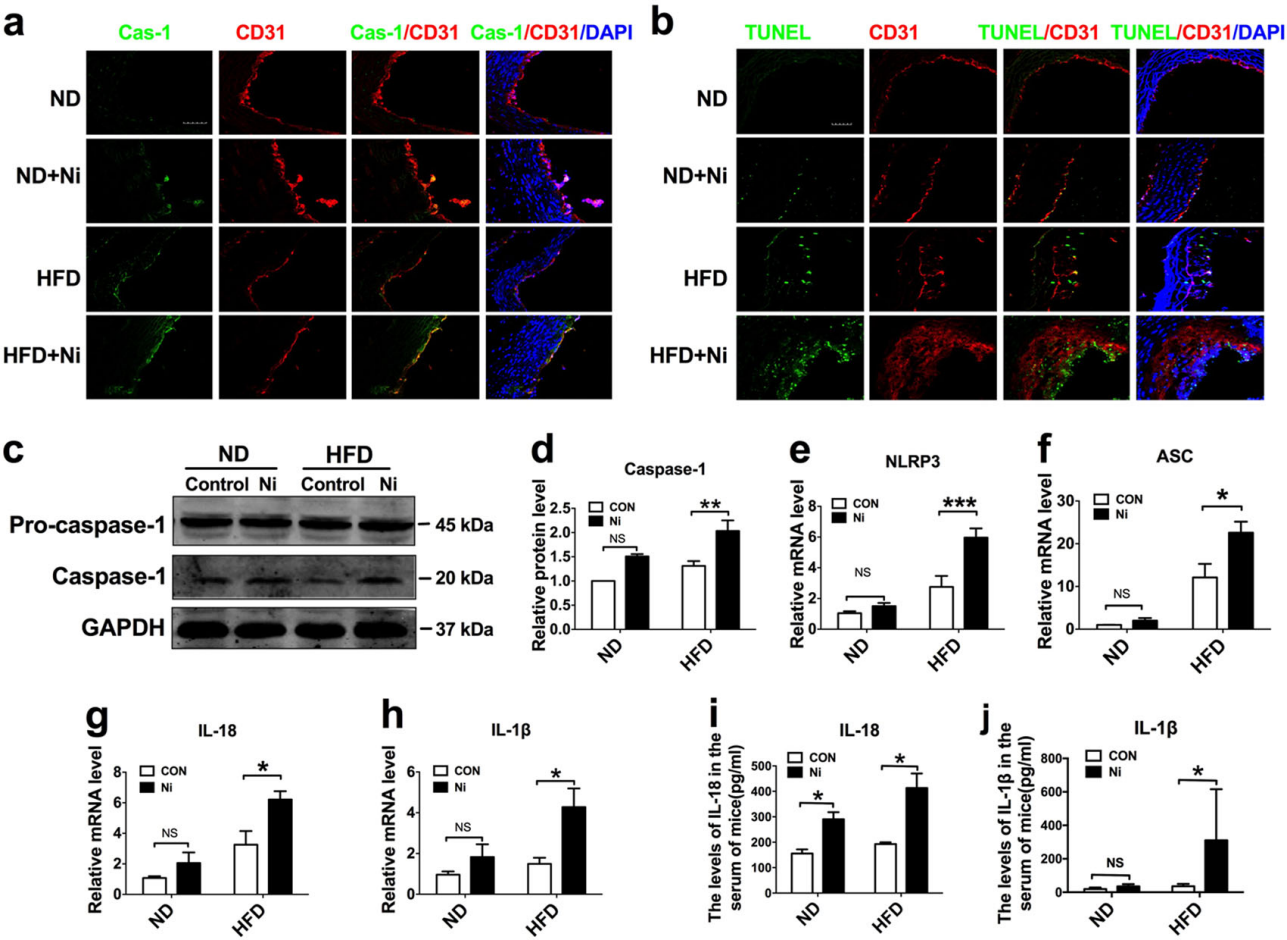
Pyroptosis of endothelial cells in the aorta of nicotine-induced ApoE^−/−^ mice. **a** Comparison of expression and subcellular distribution of caspase-1 by double staining of caspase-1 (green) and CD31 (red) in atherosclerotic lesions of ApoE^−/−^ mice among various groups: ND, ND + Ni (nicotine), HFD, or HFD + Ni. Note the presence of caspase-1 in endothelial cells as indicated by the co-localization of caspase-1 and CD31 (an endothelial marker). Magnification: ×200. **b** Identification of endothelial cell death by co-localized staining of CD31 (red) and TUNEL (green). The nuclei were stained blue with DAPI. Scale bar = 100 μm. Magnification: ×200. **c** Increases in the protein levels of caspase-1 by nicotine in the HFD group, as revealed by western blot analysis. **d**–**h** Increases in the expression of pyroptosis-related genes (NLRP3, ASC, caspase-1, IL-1β, and IL-18) at both protein and mRNA levels by nicotine in intimal samples of ApoE^−/−^ mice of the HFD group. *n* = 6 mice for each group. The data are presented as the mean ± SEM., *P < 0.05. **i**–**j** Elevation of mean serum concentrations of IL-1β and IL-18 by nicotine in HFD-fed mice, as determined by ELISA assay. The data are shown as mean ± SEM. *n* = 6 mice in each group. *P < 0.05

Pyroptosis is uniquely dependent on the activation of caspase-1, which can process cytokines IL-1β and IL-18 into their active forms and then induce pyroptotic cell death. Caspase-1 activation requires a protein platform named inflammasome, among which the NLRP3 inflammasome is the mostly studied one. We performed CD31/caspase-1 double staining and CD31/TUNEL double staining in ECs isolated from aortic arch of mice. The results in Fig. 2a, b showed that the expression level of caspase-1 and TUNEL-positive cells were both remarkably increased in the presence of nicotine in mice fed with either HFD or ND. Western blot results further showed that caspase-1 activation was enhanced in nicotine-treated HFD mice (Fig. 2c). Next, to characterize whether the expression of pyroptosis-related genes was altered in aortic intima, we isolated intimal RNA from the aorta for real-time RT–PCR analysis. We also detected EC-specific marker gene CD31 and smooth muscle cell specific marker smMHC. The results in Supplementary Figure 1 showed that expression of CD31 was abundantly enriched in the intima, but almost undetectable in the media plus adventitia. Conversely, the expression of smMHC was much lower in the intima and higher in the media plus adventitia, indicating that the isolated RNA was mainly from endothelial cells. Notably, as shown in Fig. 2d–h, the expression of NLRP3, ASC, Caspase-1, IL-1β, and IL-18 in the intimal RNA samples were significantly increased in nicotine-treated mice fed with HFD. In mice fed with ND, these genes expression also showed an upward trend upon nicotine treatment. Consistently, similar changes of serum concentrations of IL-1β and IL-18 were observed in nicotine treated and untreated mice (Fig. 2i, j).

To further validate the effects of NLRP3 activation on nicotine-induced atherosclerotic formation and ECs pyroptosis, we assessed whether administration of lentivirus that carries NLRP3 shRNA could affect atherosclerotic lesions. After 12 weeks of HFD and nicotine administration, the mice of the NLRP3 shRNA group displayed decreased atherosclerotic lesion size and less lipid deposition in aortic root compared with the mice without NLRP3 shRNA administration and the mice administrated with lenti-scramble (Supplementary Figure 2). Additionally, we found that caspase-1 dependent cell death and pyroptosis-related genes expression were reduced in the mice injected with lenti-shNLRP3 during the administration of HFD and nicotine (Supplementary Figure 3).

### Nicotine treatment triggers pyroptosis in HAECs

**Fig. 3:**
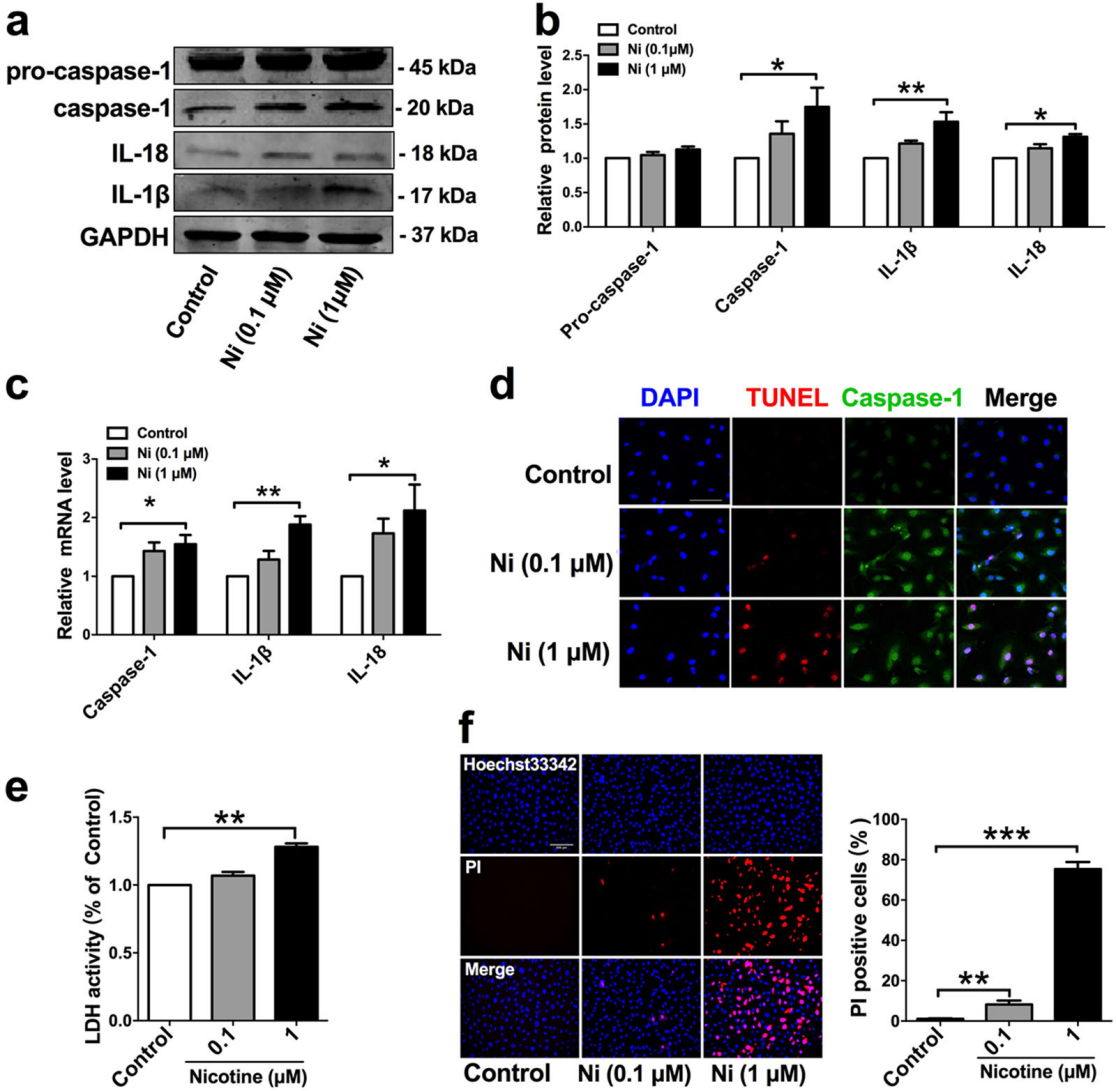
Nicotine triggered pyroptosis in human aortic endothelial cells (HAECs). **a**–**b** The protein levels of Caspase-1, IL-18, and IL-1β were upregulated in HAECs after treatment with nicotine for 24 h, as indicated by western blot results. GAPDH was used as an internal control. **c** The relative mRNA levels of pro-inflammatory cytokines (IL-1β, IL-18) and Caspase-1 were upregulated in HAECs after treatment with nicotine for 24 h. *P < 0.05, **P < 0.01. The data are represented as mean ± SEM (*n* = 3–5). **d** Caspase-1 (green) and TUNEL (red) double-positive cells were increased in the presence of nicotine. The nuclei were stained blue with DAPI. Magnification: ×200. Scale bar = 100 μm. **e** The relative LDH release was elevated in nicotine-treated endothelial cells (*n* = 3). **P < 0.01. **f** The percentage of PI (red) positive cells were increased in HAECs after treatment with nicotine (left: the representative photographs, right: the quantification of PI positive cells). Magnification: ×200. Scale bar = 500 μm. **P < 0.01, ***P < 0.001. The data are represented as mean ± SEM (*n* = 4)

To elucidate the relationship between nicotine and pyroptosis, we used HAECs for in vitro experiments. In ECs incubated with 0.1 or 1 μM nicotine, the expression of caspase-1 and pro-inflammatory cytokines (IL-1β and IL-18) were significantly increased (Fig. 3a–c). In order to further characterize nicotine-induced pyroptosis of ECs, we double stained caspase-1 and TUNEL in HAECs. The results in Fig. 3d showed that both caspase-1 activity and TUNEL-positive cells were increased in nicotine-treated HAECs. To discriminate between that apoptotic and pyroptotic cell death in the TUNEL-positive cells, we went on to conduct LDH release assay and PI staining. During pyroptosis, pores can be formed in the cell membrane and lead to the release of cellular contents and positive staining of dead cells, which can be determined by LDH release assay and PI staining, respectively^9^. Our results showed that nicotine-induced pore formation and membrane rupture, as indicated by the increased LDH activity and the extensive PI positive staining cells (Fig. 3e, f).

**Fig. 4:**
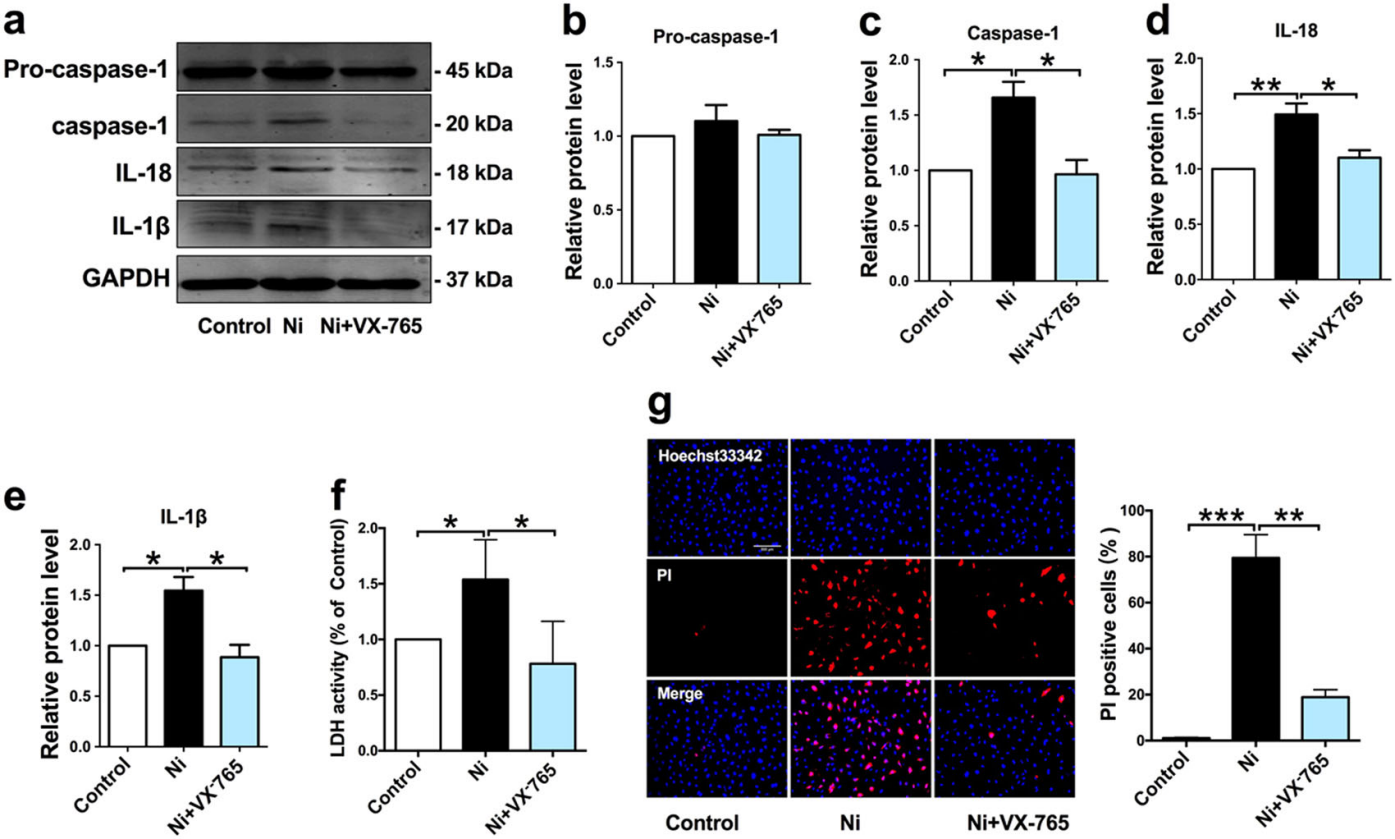
Caspase-1 inhibitor represses nicotine-induced endothelial cells pyroptosis. HAECs were pretreated with caspase-1 inhibitor (VX-765, 10 μM) for 1 h, and then the cells were incubated with nicotine (1 μM) for 24 h. **a** VX-765 inhibited the protein expression of pro-caspase-1, caspase-1, IL-18, and IL-1β in nicotine-treated endothelial cells. **b**–**e** Quantitative analysis of pyroptosis-associated protein expression. GAPDH was used as an internal control (*n* = 4). **f** Pyroptotic cell death was determined by LDH release, and the relative LDH activity was inhibited by VX-765 (*n* = 5). **g** Double staining of PI (red) and Hoechst 33342 (blue) (left: the representative photographs, right: the quantification of PI positive cells). The increased percentage of PI positive cells in nicotine treated endothelial cells were reduced after pretreatment with VX-765. The data are represented as mean ± SEM (*n* = 4). Magnification: ×200. *P < 0.05, **P < 0.01, ***P < 0.001

Next, to further investigate whether nicotine-induced pyroptosis of ECs is caspase-1 dependent, we carried out caspase-1 inhibitory experiment. Our results showed that caspase-1 selective inhibitor (VX-765) decreased the level of activated caspase-1, and inhibited the maturation of IL-18 and IL-1β (Fig. 4a–e). The cell lysis and pyroptotic cell death were reversed by VX-765, as demonstrated by the reduction of LDH release and the percentage of PI positive cells (Fig. 4f, g).

### NLRP3-ASC inflammasome is involved in nicotine-induced EC pyroptosis

**Fig. 5:**
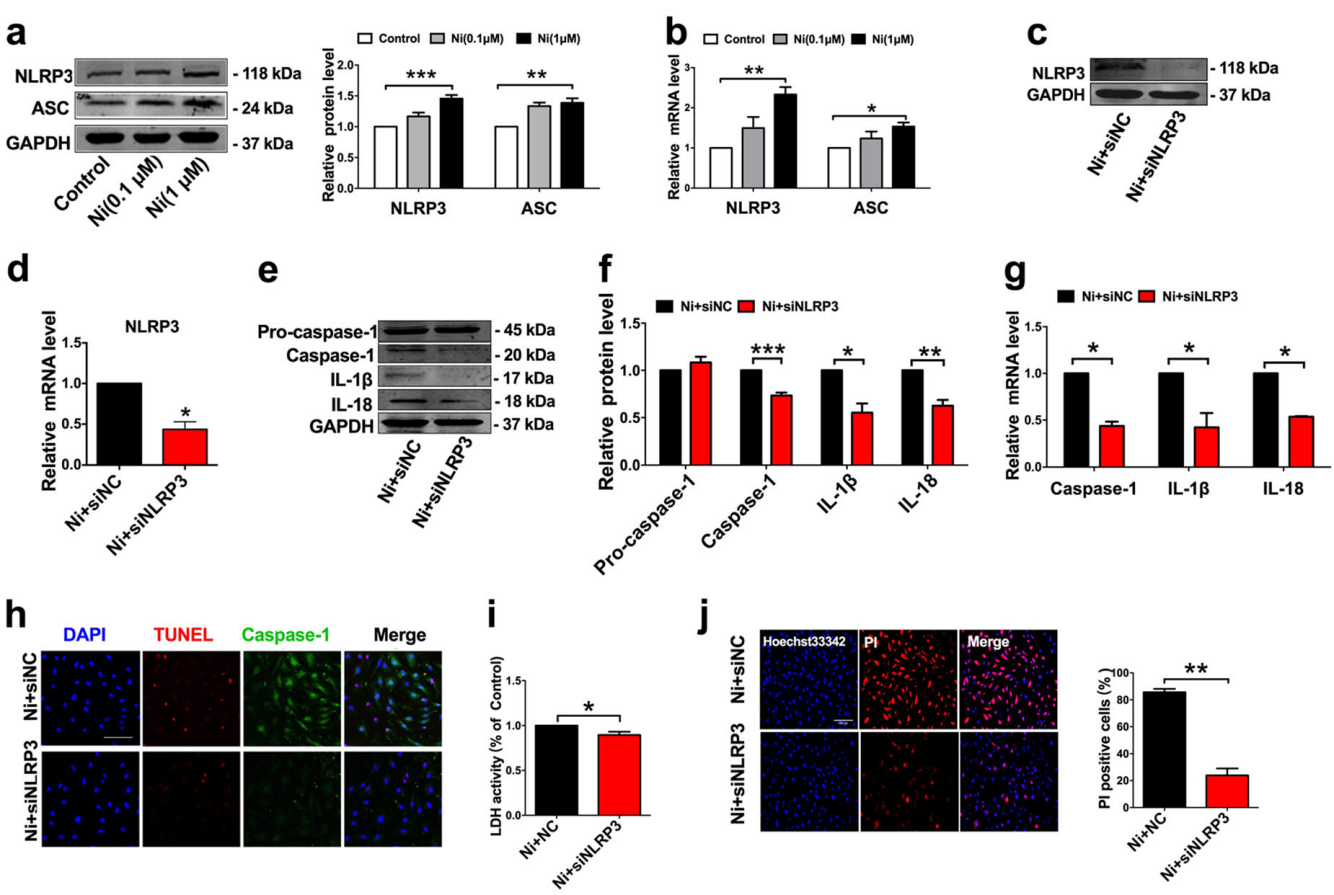
NLRP3 inflammasome is involved in nicotine-induced endothelial cells pyroptosis. **a** The expression levels of NLRP3 and ASC were increased in HAECs treated with nicotine (0.1, 1 μM) for 24 h, as determined by western blot results. **b** The mRNA levels of NLRP3 and ASC were increased in HAECs treated with nicotine (0.1, 1 μM) for 24 h. **c**–**d** NLRP3 siRNA silenced the expression of NLRP3 at both protein and mRNA level. **e**–**f** The protein levels of Caspase-1, IL-1β, and IL-18 in nicotine-treated HAECs were inhibited after transfection with siNLRP3. NC indicates negative control (*n* = 4). **g** The mRNA levels of caspase-1, IL-1β, and IL-18 were inhibited by siNLRP3 in the presence of nicotine (*n* = 4). *P < 0.05, **P < 0.01, ***P < 0.001. **h** TUNEL (red) and caspase-1 (green) double-positive cells were decreased in nicotine-treated endothelial cell that transfected with siNLRP3. The nuclei were stained blue with DAPI. Magnification: ×200. Scale bar denotes 100 μm. **i** Pyroptotic cell death was determined by LDH release, and the relative LDH activity was suppressed by siNLRP3 in the presence of nicotine (*n* = 3). *P < 0.05. **j** The percentage of PI positive cells was declined after transfection with siNLRP3 in nicotine-treated HAECs (left: the representative photographs, right: the quantification of PI positive cells). Magnification: ×200. Scale bar = 500 μm. **P < 0.01. The data are represented as mean ± SEM (*n* = 4)

Increases in the protein levels of cleaved caspase-1 (Casp1 p20) and mature IL-1β (17 kDa) and mature IL-18 (18 kDa) are the hallmarks of NLRP3 inflammasome activation^19^. NLRP3 recruits caspase-1 through ASC, allowing activated caspase-1 to cleave pro-IL-1β to mature IL-1β. We therefore measured the expression levels of NLRP3 and ASC in HAECs after treatment with nicotine. As depicted in Fig. 5a, b, NLRP3 and ASC were remarkably upregulated by nicotine at a concentration of 1 μM.

**Fig. 6:**
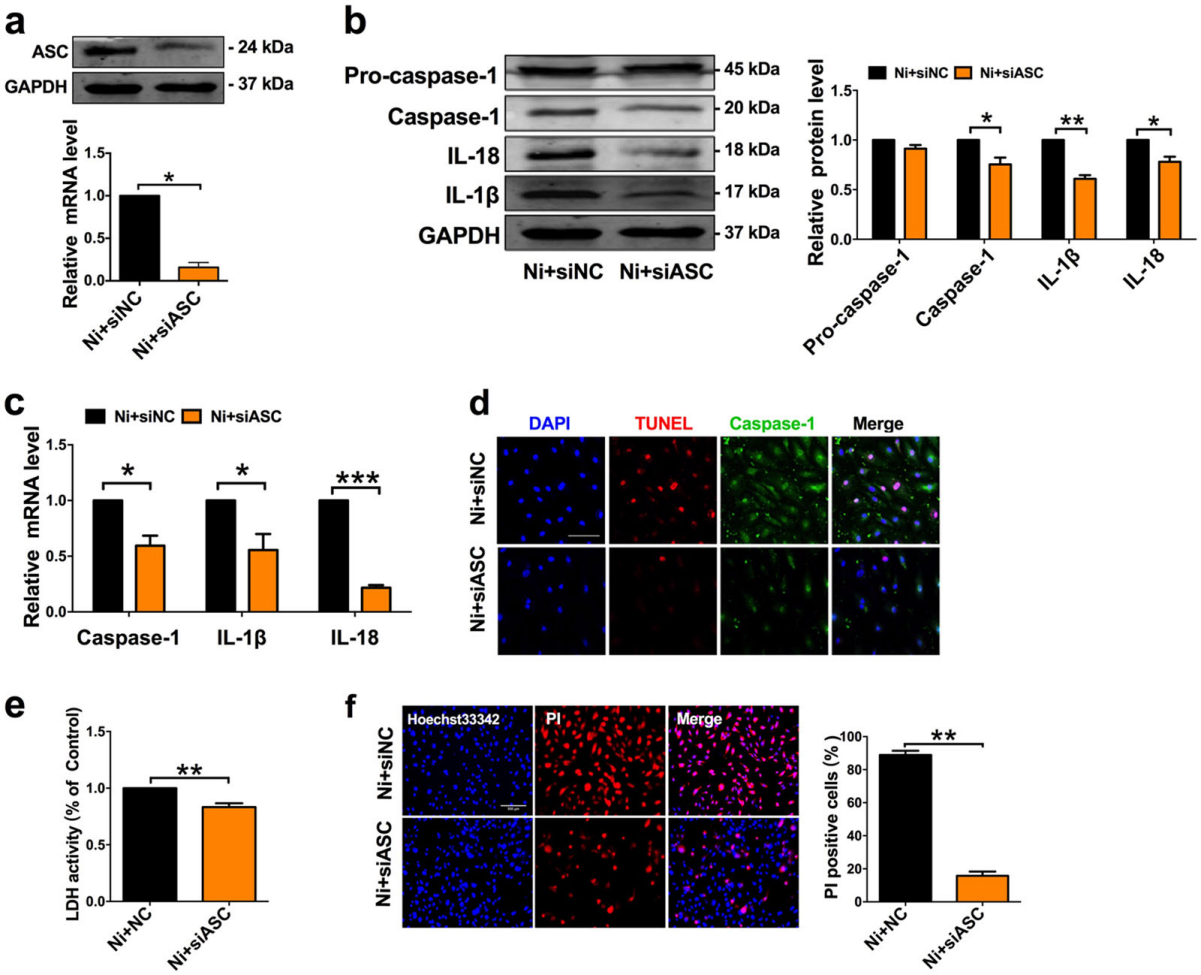
ASC deficiency blocked nicotine-induced endothelial cells pyroptosis. **a** Verification of silencing efficiency of ASC by siRNA in nicotine-treated HAECs. **b** The protein levels of Caspase-1, IL-1β, and IL-18 in nicotine-treated HAECs were decreased after transfection of siASC. NC indicates negative control. **c** The mRNA levels of Caspase-1, IL-1β, and IL-18 in nicotine-treated HAECs were decreased after transfection of siASC. (*n* = 4). *P < 0.05, **P < 0.01, ***P < 0.001. **d** TUNEL (red) and caspase-1 (green) double-positive cells were reduced by siASC in the presence of nicotine. Magnification: ×200. Scale bar denotes 100 μm. **e** Pyroptotic cell death was determined by LDH release, and the relative LDH activity was suppressed by ASC siRNA (*n* = 3). **P < 0.01. **f** The percentage of PI positive cells was declined after transfection with siASC in nicotine-treated HAECs. (left: the representative photographs, right: the quantification of PI positive cells). Magnification: ×200. Scale bar = 500 μm. **P < 0.01. The data are represented as mean ± SEM (*n* = 4)

Moreover, the results in Fig. 5c, d clearly indicated the presence of NLRP3 in HAECs. Silencing of NLRP3 reduced the activation of caspase-1 and the production of IL-1β and IL-18 (Fig. 5e–g). Furthermore, silencing of NLRP3 dampened the ability of nicotine to induce HAECs pyroptosis, as evidenced by the decreases in the number of TUNEL and caspase-1 double-positive cells (Fig. 5h). Silencing of NLRP3 also abrogated the release of LDH and the increase of PI positive cells, indicating an inhibition of the cell lysis and pyroptotic cell death by nicotine (Fig. 5i, j). In addition, ASC silencing also significantly suppressed the expression of caspase-1 and the production of pro-inflammatory cytokines (IL-1β and IL-18), along with inhibition of caspase-1-dependent cell death (Fig. 6a–f).

### Nicotine-induced endothelial cells pyroptosis requires ROS

**Fig. 7:**
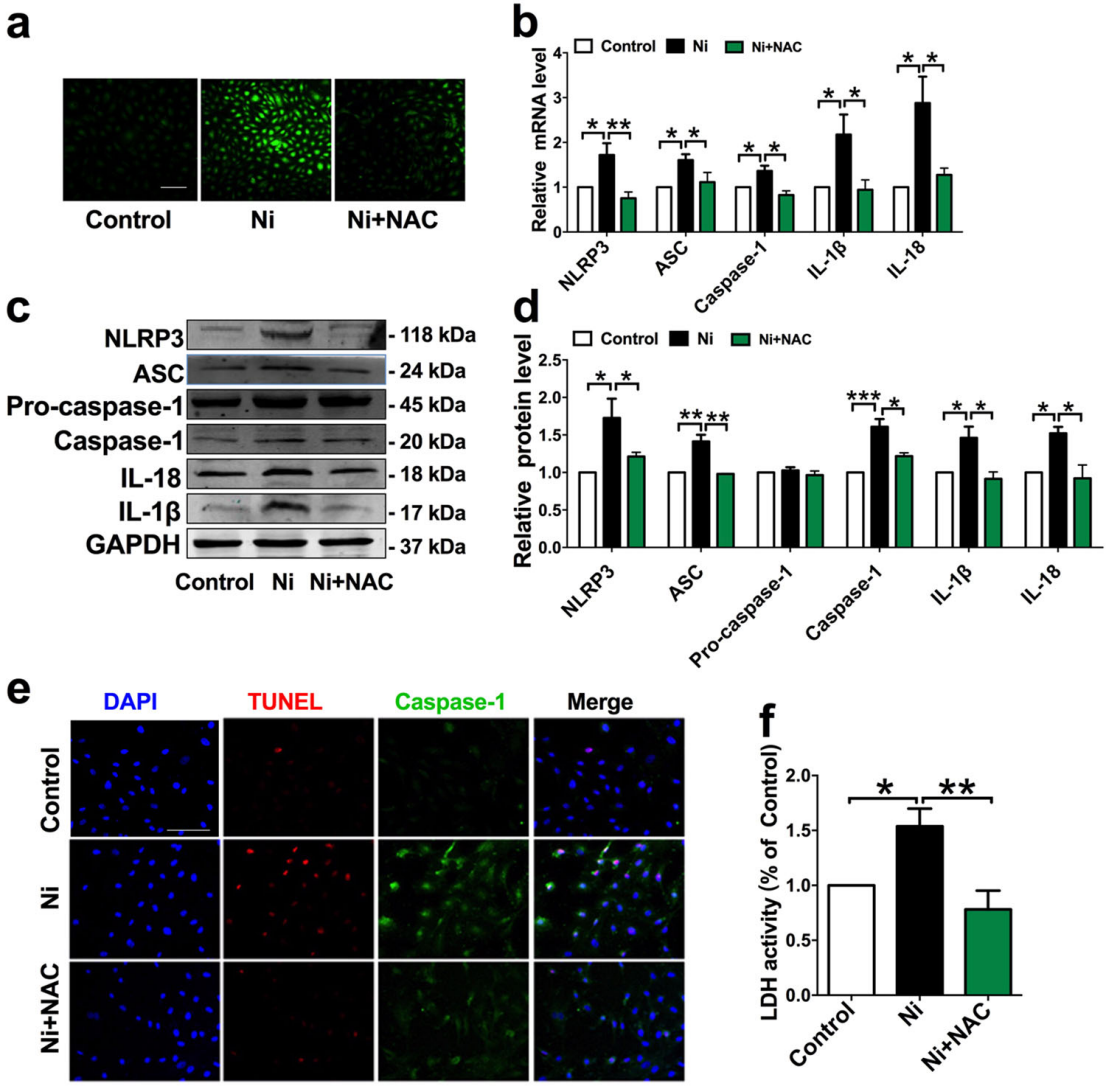
Pretreatment with ROS scavenger mitigated nicotine-induced NLRP3 inflammasome activation and endothelial cells pyroptosis. **a** Nicotine increased intracellular ROS level of HAECs, and this increase was inhibited by N-acetyl-cysteine (NAC, 5 mM). **b** Nicotine-induced the upregulation of NLRP3, ASC, Caspase-1, IL-1β, and IL-18 mRNA levels were decreased by NAC (*n* = 3–5). **c**–**d** NAC pretreatment suppressed the upregulation of Caspase-1, IL-1β, and IL-18 protein levels in the presence of nicotine. *P < 0.05, **P < 0.01, ***P < 0.001. **e** TUNEL (red) and Caspase-1 (green) double-positive cells were decreased when pretreatment with NAC. Scale bar indicates 100 μm. Magnification: ×200. **f** Pyroptotic cell death was determined by LDH release, and the relative LDH activity was decreased when pretreated with NAC (*n* = 5). *P < 0.05, **P < 0.01

Reactive oxygen species (ROS) are essential for inflammasome activation^20^. To elucidate the possible role of ROS in nicotine-induced pyroptosis, we first detected the changes of ROS level in ApoE^−/−^ mice fed with nicotine. Our results demonstrated that nicotine treatment dramatically increased ROS levels in ApoE^−/−^ mice fed with HFD (Supplementary Figure 4). Treatment of HAECs with nicotine fostered intracellular ROS production, and this was buffered by N-acetyl-cysteine (NAC), a ROS inhibitor (Fig. 7a). NAC also inhibited nicotine-induced gene expression of inflammasome components and inflammatory cytokines, including NLRP3, ASC, Caspase-1, IL-1β, and IL-18 (Fig. 7b), and the protein levels of NLRP3, ASC, cleaved caspase-1 (Casp1 p20), and mature IL-1β and mature IL-18 were all blocked by NAC (Fig. 7c, d), suggesting that NLRP3 inflammasome activation was dependent on ROS generation. Additionally, NAC pretreatment reduced the number of TUNEL and Caspase-1 double-positive cells (Fig. 7e), and LDH activity in nicotine treated ECs (Fig. 7f), suggesting the importance of ROS in nicotine-induced EC pyroptosis.

## Discussion

**Fig. 8:**
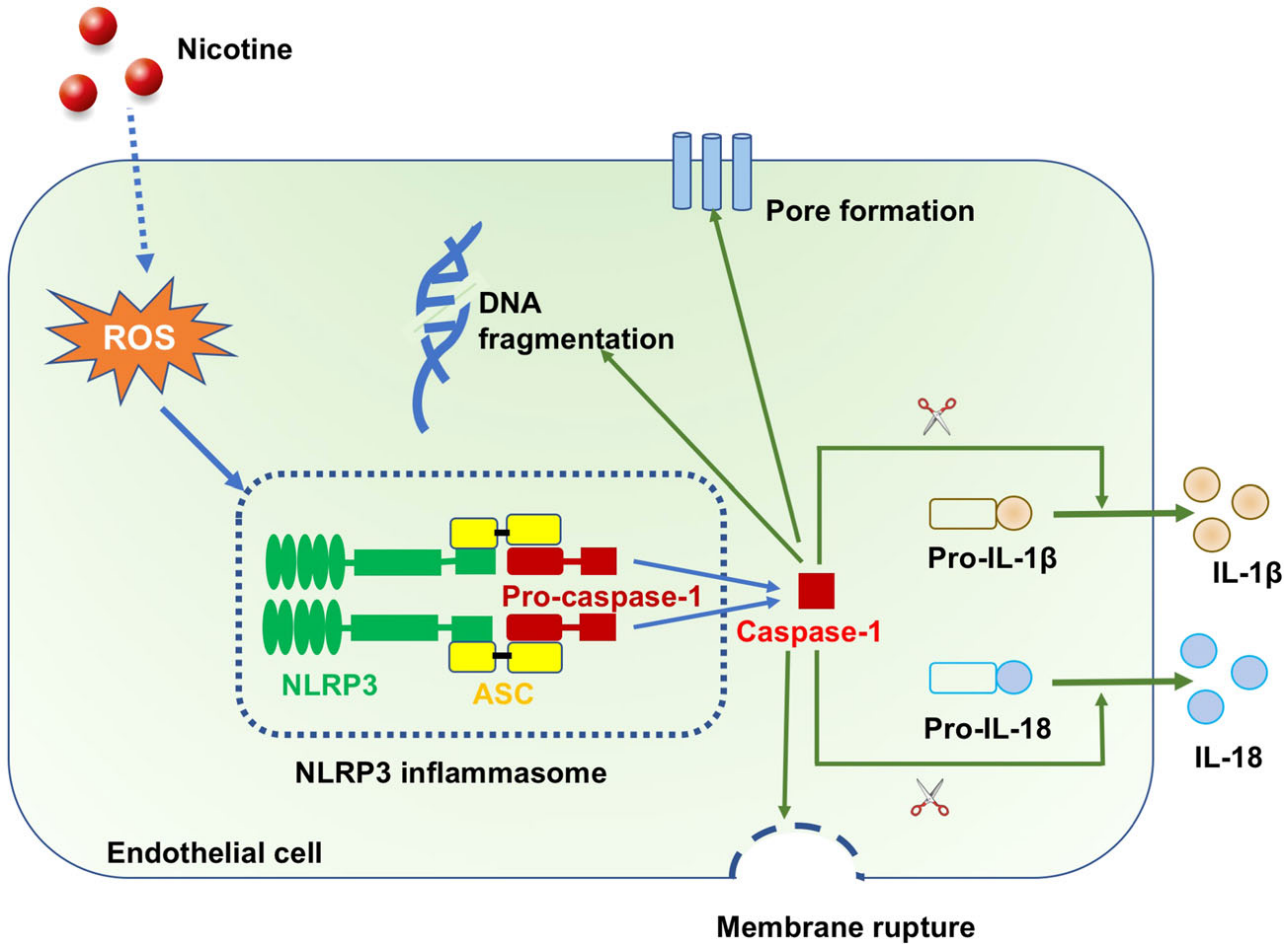
Proposed model of endothelial pyroptosis in nicotine-promoted atherosclerosis. Nicotine enters endothelial cell, and induces the production of ROS, which activates NLRP3 inflammasome, leading to the activation of caspase-1. Activated caspase-1 triggers pore formation of membrane, DNA fragmentation and release of mature IL-1β and IL-18 from cells, causing a sterile inflammation response, further contributing to the pyroptotic cell death and subsequent promoting atherosclerosis

The data presented in the current study demonstrated that nicotine-induced NLRP3 inflammasome activation, inflammatory response, and subsequent pyroptosis in the setting of atherosclerosis. Silencing of NLRP3 inflammasome or reducing ROS production inhibited nicotine-induced caspase-1 activation, inflammatory cytokines (IL-1β and IL-18) secretion, and pyroptotic death of endothelial cells (Fig. 8). Our study unraveled pyroptotic cell death as a cellular mechanism for the pro-atherosclerotic property of nicotine, thereby advancing our understanding of the pathophysiology of nicotine, cigarette smoking, and consumption of other tobacco products.

Several risk factors of atherosclerosis have been identified including hyperlipidaemia, hypertension, diabetes, smoking, and aging. It has been demonstrated that cigarette smoking promotes the development of atherosclerotic plaques^21–23^. Nicotine is one of effective components of cigarettes. Experimental studies have observed that nicotine intake promotes the development of atherosclerosis^18,24^. Consistently, our study showed that nicotine treatment promoted atherosclerosis in ApoE^−/−^ mice fed with HFD, as indicated by the larger lesion area and more lipids content. In mice fed with ND, nicotine also increased atherosclerotic lesion area and lipid content; nevertheless, this effect was not as great as those in mice fed with HFD. The possible explanation for this difference may be related to the exposure duration and concentration of nicotine.

Atherosclerosis is a complex inflammatory disease of medium and large-sized arteries, in which three cellular types are mainly involved, including endothelial cells, macrophages, and smooth muscle cells^25–27^. The pathophysiologic mechanisms by which nicotine promotes vascular diseases particularly atherosclerosis is manifold and complex. A previous study reported that nicotine activates bone marrow–derived mast cells (MCs) via α7 nicotinic acetylcholine receptor (α7nAChR), and mast cell (MC) deficiency prevents nicotine-induced plaque formation and composition change, implying the important role of MC in nicotine-enhanced atherogenesis in ApoE^−/−^ mice^18^. Another study demonstrated that nicotine has an enhancing effect on atherosclerosis in the LDLR^−/−^ mouse model via increasing the production of pro-inflammatory cytokines by macrophages^16^. In agreement with previous study, our study on mouse macrophages showed that nicotine elevated the pyroptosis-related proteins expression and LDH activity, but caspase-1 was not obviously and totally merged with CD68 (a marker of macrophage) in the in vivo aortic tissue of ApoE^−/−^ mice (Supplementary Figure 5), indicating that the activation of caspase-1 exists beyond macrophages. Therefore, we suspected whether endothelial cells also undergo inflammatory response in nicotine-induced atherosclerosis. Endothelial cells play a critical role in maintaining the integrity of vessel wall, and endothelial cell injury is an initiator of atherosclerosis^28^ and has been documented in cigarette smokers^28^. Moreover, nicotine has also been shown to participate in vascular inflammation and endothelial dysfunction^29, 30^.

Based on this information, we are pushed to explore the role of endothelial cells in nicotine-induced plaque formation. Cell death and inflammation are fundamental characteristics in the initiation and development of atherosclerosis. Pyroptosis is an inflammatory form of cell death and has been implicated in cardiovascular diseases^31, 32^. In this study, we found that endothelial cells displayed characteristic features of pyroptosis in the aorta of nicotine-treated ApoE^−/−^ mice fed with HFD, as evidenced by increased levels of caspase-1, IL-1β, IL-18, NLRP3, ASC, and TUNEL-positive cells. Differing from caspase-3 dependent apoptosis, pyroptosis requires the activation of caspase-1^9^. Once activated, caspase-1 executes its function to process the precursor of the inflammatory cytokines IL-1β and IL-18, to their mature forms^8^. We observed that in the presence of nicotine, endothelial cells had increased levels of active forms of Caspase-1, IL-1β, and IL-18, and increased numbers of caspase-1 dependent cell death, indicating that nicotine-induced endothelial cell pyroptosis might be a cellular mechanism for the development of atherosclerosis.

Activation of caspase-1 during pyroptosis required a protein platform called inflammasome^33^. Among these inflammasomes, NLRP3 is the most extensively studied. Upon activation, NLRP3 recruits ASC (apoptotic speck like protein containing a CARD domain) and promotes the activation of caspase-1, thus processing inflammatory cytokines to their mature forms ultimately leading to pyroptotic cell death^34^. NLRP3 inflammasome has been reported to play an important role in inflammation-associated liver fibrosis and atherogenesis^35,36^. Various cellular cues, such as cholesterol crystals, calcium phosphate crystals, and oxidized low-density lipoprotein can activate NLRP3 inflammasome in macrophages^37^. However, little is known about NLRP3 in HAECs. We found here that the expression of both NLRP3 and ASC was upregulated in nicotine-treated endothelial cells. In contrast, when silencing NLRP3 or ASC in endothelial cells by RNA interference experiment, these cells became immune to nicotine-induced pyroptotic death, suggesting that NLRP3 inflammasome is required for nicotine-induced pyroptosis in HAECs.

Generation of ROS is a common upstream mechanism implicated in NLRP3 inflammasome activation^20, 38^. In agreement with this view, our study showed that nicotine-promoted ROS production and this oxidative stress may likely be an upstream mechanism for the activation of NLRP3 inflammasome activation, which can be neutralized by N-acetyl-cysteine (NAC), a ROS inhibitor. As the antioxidant agent NAC diminished both inflammasome activation and inflammatory cytokine maturation, and subsequently pyroptotic death induced by nicotine in HAECs.

Collectively, our results provide the first evidence that the pro-atherosclerotic property of nicotine is primarily conferred by its ability to stimulate ROS production, activate NLRP3 and caspase-1, and ultimately result in pyroptosis of endothelial cells. Hence, pyroptosis is likely a cellular mechanism underlying the detrimental effect of nicotine on atherosclerosis with the production of ROS and activation of NLRP3 as the upstream mediators. Targeting caspase-1 dependent pyroptosis therefore might be considered a new approach for alleviating atherosclerotic lesions induced by nicotine.

## Materials and methods

### Animals and ethics statements

The ApoE^−/−^ mice were kept at standard house conditions with temperature of 23 ± 1 °C and humidity of 55–60%. The mice were randomly divided into four groups (*n* = 6 for each group): ND group, ND + Ni group, HFD group, and HFD + Ni group (ND-normal diet, HFD-high-fat diet, Ni-nicotine). The composition of HFD includes 10% lard, 4% milk powder, 2% cholesterol, and 0.5% sodium cholate. The mice (eight-weeks old) were administrated with the water containing 100 μg/mL nicotine for 12 weeks. After 12 weeks of treatment, the mice (20 weeks old) were anesthetized and killed for measurements of aortic lesion size and biochemical analysis. Our study protocol was approved by the ethic committees of Harbin Medical University and the experimental procedures were in accordance with the Guide for the Care and Use of Laboratory Animals published by the US National Institutes of Health (NIH Publication No. 85–23, revised 1996).

### Lentivirus injection

The lentiviral vectors carrying a short hairpin RNA (shRNA) for NLRP3 (shNLRP3) and a negative control shRNA (shControl) were designed and chemically synthesized by Hanyin Biotechnology Limited Company (Shanghai, China). The sequences of the NLRP3 shRNA were: sense, 5′-GATCCGATCTAGCCACACTCATGAAGAGAACTTTCATGAGTGTGGCTAGATCTTTTTTG-3′, and antisense, 5′-AATTCAAAAAAGATCTAGCCACACTCATGAAAGTTCTCTTCATGAGTGTGGCTAGATCG-3′. The constructs were diluted to a total volume of 300 μL containing 4 × 10^7^ TU (transducing units) and administered into the mice fed with HFD and nicotine through tail vein injection.

### Histology and immunohistochemistry

The aortic roots were isolated from mice, fixed with 4% paraformaldehyde, embedded in optimum cutting temperature compound (OCT), and cut into 5 µm-thick sections. Atherosclerotic lesions were detected by hematoxylin and eosin (HE) staining assay, and the lipid deposition was evaluated by Oil Red O staining kit (Nanjing Jiancheng Biology Engineering Institute, Nanjing, Jiangsu, China), as previously described^39^. TUNEL staining and immunostaining of caspase-1 and CD31 (endothelial cell marker) on the aortic roots were performed to detect cell death and activation of caspase-1 in endothelial cells. The images were examined under a laser scanning confocal microscope (FV300, Olympus, Japan).

### Enzyme-linked immunosorbent assay (ELISA)

Serum samples were collected from ApoE^−/−^ mice after treatment with nicotine for 3 months. Serum concentrations of IL-1β and IL-18 in ApoE^−/−^ mice were determined by ELISA kit (Wuhan Boster Biological Technology, Ltd., Wuhan, Hubei, China) according to the manufacturer’s instructions.

### Cell culture and transfection

Human aortic endothelial cells (HAECs) were obtained from ScienCell Research Laboratories (Carlsbad, CA, USA). The cells were grown in Endothelial Cell Medium (ECM, ScienCell Research Laboratories, Carlsbad, CA, USA) supplemented with 10% FBS, 1% (v/v) penicillin/streptomycin, and 1% endothelial cell growth factors at 37 °C with 5% CO_2_ and 95% air. Nicotine and total ROS scavenger N-acetyl-l-cysteine (NAC) were purchased from Sigma-Aldrich. siRNA of NLRP3 and ASC and the negative control were synthesized by GenePharma (Shanghai, China). The sequences of the NLRP3 siRNA used in our experiments are sense, 5′-CAACAGGAGAGACCUUUAUTT-3′, and antisense, 5′-AUAAAGGUCUCUCCUGUUGTT-3′. The sequences of the ASC siRNA are sense, 5′-UCGCGAGGGUCACAAACGUTT-3′, and antisense, 5′-ACGUUUGUGACCCUCGCGATT-3′. For the transfection of siNLRP3 and siASC, Opti-MEM medium (Invitrogen, CA, USA) and Lipofectamine 2000 reagent (Invitrogen CA, USA) were used. After 24 h of transfection, the medium was replaced by fresh medium containing nicotine. For experiments involving pharmacological inhibitors, endothelial cells were pretreated with NAC (5 mM) for 2 h and subsequently treated by nicotine (1 μM) for 24 h in the presence of this inhibitor.

### Detection of reactive oxygen species (ROS)

Reactive oxygen species assay kit (Beyotime, China) was used to detect the accumulation of ROS in endothelial cells according to the manufacturer’s instructions. Briefly, endothelial cells cultured on the coverslips in 24-well plates were loaded with DCFH-DA (10 μM) in serum-free medium in dark at 37 °C for 20 min, following by washes with PBS three times. Fluorescence was examined by confocal microscopy (FV300, Olympus, Japan).

### RNA isolation and RT–PCR

The detailed protocol for the isolation of intimal RNA from aorta was the same as described previously^40^. TRIZOL reagent (Invitrogen, CA, USA) was applied to extract total RNA from intima and HAECs. High-Capacity cDNA Reverse Transcription Kit (Applied Biosystems, Foster City, CA, USA) was used for the reverse transcription of extracted RNA into cDNA. The first-strand cDNA was amplified using SYBR Green I incorporation method to quantify the relative expression of mRNA on ABI 7500 fast Real-Time PCR system (Applied Biosystems, USA). After amplification, the threshold cycle (Ct) was determined and relative mRNA levels were calculated based on the 2^−△△Ct^ method. GAPDH was used as an internal control for data normalization. The sequences of the primers used are provided in Supplementary Table 1.

### Western blot analysis

Total protein was extracted from endothelial cells using the same procedures as described in detail elsewhere^41^. The protein concentrations were determined by BCA Protein Assay kit (Bio-Rad, Mississauga, ON, Canada). Equal amounts of protein lysates were separated by SDS-PAGE and transferred onto nitrocellulose membranes followed by block with 5% skimmed milk at room temperature for 2 h. Subsequently, the membranes were incubated with the primary antibodies against NLRP3 (Proteintech, Chicago, USA, 1:1000, Cat. No.: 19771-1-AP), ASC (Santa Cruz, USA, 1:500, Cat. No.: sc-22514-R), Caspase-1 (Proteintech, Chicago, USA, 1:1000, Cat. No.: 22915-1-AP), IL-1β (ABclonal, Boston, USA, 1:1000, Cat. No.: A1112), IL-18 (ABclonal, Boston, USA, 1:1000, Cat. No.: A1115), or GAPDH (Proteintech, Chicago, USA, 1:2000, Cat. No: 60004-1-lg) at 4 °C overnight. After washing with PBST three times, the membranes were incubated with the fluorescence-conjugated anti-rabbit IgG secondary antibody (1:10,000) for 1 h. Western blot bands were examined and analyzed by Odyssey Imaging System (LI-COR, Inc., Lincoln, NE, USA).

### Immunofluorescence

Immunofluorescence staining was performed to detect the expression of caspase-1 in endothelial cells. Briefly, the cells were fixed with 4% paraformaldehyde for 30 min, penetrated by 0.6% Triton X-100 for 1 h, and then blocked with goat serum. Subsequently, the cells were incubated with anti-caspase-1 antibody at 4 °C overnight, followed by incubation with Alexa Fluor-conjugated secondary antibody (Invitrogen, Carlsbad, CA, USA) in the dark for 1 h. The nuclei were stained by 4′,6-diamidino-2-phenylindole (DAPI; Beyotime, China) for 20 min. The cells were imaged under a laser scanning confocal microscope (FV300, Olympus, Japan).

### Analysis of DNA fragmentation

TUNEL staining was carried out to detect DNA fragmentation of endothelial cells as previously described^32^. Briefly, endothelial cells were cultured on coverslips in a 24-well plate. After designated treatments, they were fixed with 4% paraformaldehyde and permeabilized with 0.1% Triton X-100. Subsequently, the cells were incubated with TUNEL reaction mixture at 37 °C in the dark for 1 h, and stained by DAPI. The cells were examined under a confocal laser scanning microscope (FV300, Olympus, Japan).

### Cell death assay

Pyroptotic cell death was evaluated with LDH release assay and Hoechst 33342/PI staining. For LDH release, cell culture supernatants were collected and the LDH activity was detected using the LDH assay kit (Nanjing Jiancheng Biology Engineering Institute, Nanjing, Jiangsu, China). Briefly, 25 μL cell supernatant and 25 μL substrate were mixed together and incubated at 37 °C for 15 min. Then 25 μL 2,4-dinitrophenylhydrazine was added into the samples and incubated at 37 °C for 15 min. Finally, 250 μL 0.4 mol/L NaOH solution was added and incubated at room temperature for 5 min. The absorbance was measured at 450 nm on a spectrophotometric microplate reader. For Hoechst 33342/PI staining, HAECs (10^5^ cells/well) were cultured in a 12-well plate and were treated with test drugs or siRNA for the duration as to be specified in the appropriate section. The cells were then incubated with a mixed solution of Hoechst 33342 and PI for 25 min and photographed under a fluorescence microscope.

### Data analysis

The data are expressed as mean ± SEM. GraphPad Prism 5.0 software was used to process the data. For comparisons between two groups, unpaired *t*-test was performed; for comparisons among multiple groups, One-way analysis of variance (ANOVA) test was performed. *P* < 0.05 was considered statistically significant.

## Electronic supplementary material

Supplementary files
