# Supplementary material for: Nicotine promotes atherosclerosis via ROS-NLRP3-mediated endothelial cell pyroptosis
Source: Cell Death Dis. 2018 Feb 7;9(2):171. doi: 10.1038/s41419-017-0257-3 (PMC5833729; doi:10.1038/s41419-017-0257-3)
Supplement: Supplementary file 1 — Supplementary files [file 41419_2017_257_MOESM1_ESM.pdf]

Nicotine promotes atherosclerosis via ROS-NLRP3-mediated endothelial cells pyroptosis

Xianxian Wu[1, 2*], Haiying Zhang[1*], Wei Qi[3*], Ying Zhang[1], Jiamin Li[1], Zhange Li[1], Yuan Lin[1], Xue Bai[1], Xin Liu[1], Xiaohui Chen[1], Huan Yang[1], Chaoqian Xu[1], Yong Zhang[1,4], Baofeng Yang[1,5]

[1]Department of Pharmacology (the State-Province Key Laboratories of Biomedicine-Pharmaceutics of China, Key Laboratory of Cardiovascular Research, Ministry of Education), College of Pharmacy, Harbin Medical University, Harbin, 150081, China;

[2]Institute of Laboratory Animal Science, Chinese Academy of Medical Sciences (CAMS) & Comparative Medicine Centre, Peking Union Medical Collage (PUMC), Beijing, People's Republic China;

[3]Department of Inorganic Chemistry and Physical Chemistry, College of Pharmacy, Harbin Medical University, Harbin, 150081, China;

[4]Institute of Metabolic Disease, Heilongjiang Academy of Medical Science, Harbin, 150086, China;

[5]Department of Pharmacology and Therapeutics, Melbourne School of Biomedical Sciences, Faculty of Medicine, Dentistry and Health Sciences, The University of Melbourne, Melbourne, 3010, Australia.

*These authors contributed equally to this work.

Correspondence and requests for materials should be addressed to Yong Zhang (email: hmuzhangyong@hotmail.com) or Baofeng Yang (email: yangbf@ems.hrbmu.edu.cn)

Supplementary table 1. Primers used for real-time RT-PCR (h and m indicate human and mouse species, respectively).

Gene	Primer Sequences (forward, reverse)
CD31(m)	F: 5'-ACGCTGGTGCTCTATGCAAG-3'<br>R: 5'-TCAGTTGCTGCCCATTCATCA-3'
NLRP3 (h)	F: 5'-CACCTGTTGTGCAATCTGAAG-3'<br>R: 5'-GCAAGATCCTGACAACATGC-3'
NLRP3(m)	F: 5'-ATTACCCGCCCGAGAAAGG-3'<br>R: 5'-TCGCAGCAAAGATCCACACAG-3'
ASC (h)	F: 5'-AGGCCTGCACTTTATAGACC-3'<br>R: 5'-GCTGGTGTGAAACTGAAGAG-3'
ASC (m)	F: 5'-CTTGTCAGGGGATGAACTCAAAA-3'<br>R: 5'-GCCATACGACTCCAGATAGTAGC-3'
Caspase-1(h)	F: 5'-CCTTAATATGCAAGACTCTCAAGGA-3'<br>R: 5'-TAAGCTGGGTTGTCCTGCACT-3'
Caspase-1(m)	F: 5'-CTTGGAGACATCCTGTCAGGG-3'<br>R: 5'-AGTCACAAGACCAGGCATATTCT-3'
IL-1β (h)	F: 5'-TACCTGTCCTGCGTGTTGAA-3'<br>R: 5'-TCTTTGGGTAATTTTTGGGATCT-3'
IL-1β (m)	F: 5'-GCAACTGTTCCTGAACTCAACT-3'<br>R: 5'-ATCTTTTGGGGTCCGTCAACT-3'
IL-18 (h)	F: 5'-TGCATCAACTTTGTGGCAAT-3'<br>R: 5'-ATAGAGGCCGATTTCCTTGG-3'
IL-18 (m)	F: 5'-GACTCTTGCGTCAACTTCAAGG-3'<br>R: 5'-CAGGCTGTCTTTTGTCAACGA-3'
GAPDH (h, m)	F: 5’-AAGGTCGGAGTCAACGGATTT-3’<br>R: 5’- AGATGATGACCCTTTTGGCTC-3’

Supplementary Figures

Supplementary Figure 1

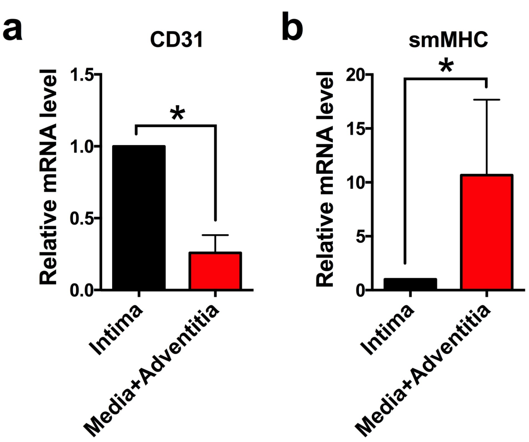

Figure S1. Confirmation of endothelial cell purity in isolated intima. (a) Endothelial cell marker CD31 expression was examined by Real-time RT-PCR analysis in intima and media plus adventitia. (b) Smooth muscle marker smMHC was examined by Real-time RT-PCR analysis in intima and media plus adventitia. n = 4 mice. **P*< 0.05.

Supplementary Figure 2

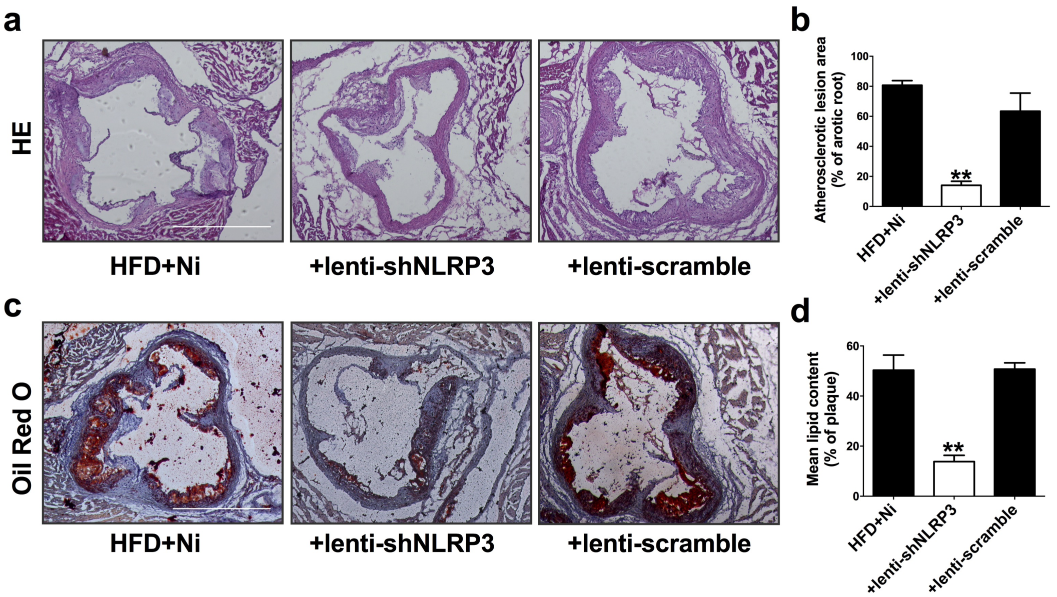

Figure S2. NLRP3 knockdown inhibits atherosclerotic lesion formation in the mice administrated with HFD and nicotine. (a) HE staining of aortic root sections showing the atherosclerotic lesions in $ApoE^{-/-}$ mice injected with lentivirus carrying NLRP3 shRNA or scramble shRNA while administrated with 12-week high-fat diet (HFD) and nicotine(Ni). Magnification: 40×. Scale bar indicates 2000 μm. (b) Quantification of the lesion area per section in different groups. n = 6 mice in each group. (c) Oil red O

staining of aortic root sections showing the lipid deposition in atherosclerotic lesions. Scale bar indicates 2000 μm. Magnification: 40×. (d) Quantification of lipid content. n = 6 mice in each group. $**P< 0.01$ vs HFD+Ni.

Supplementary Figure 3

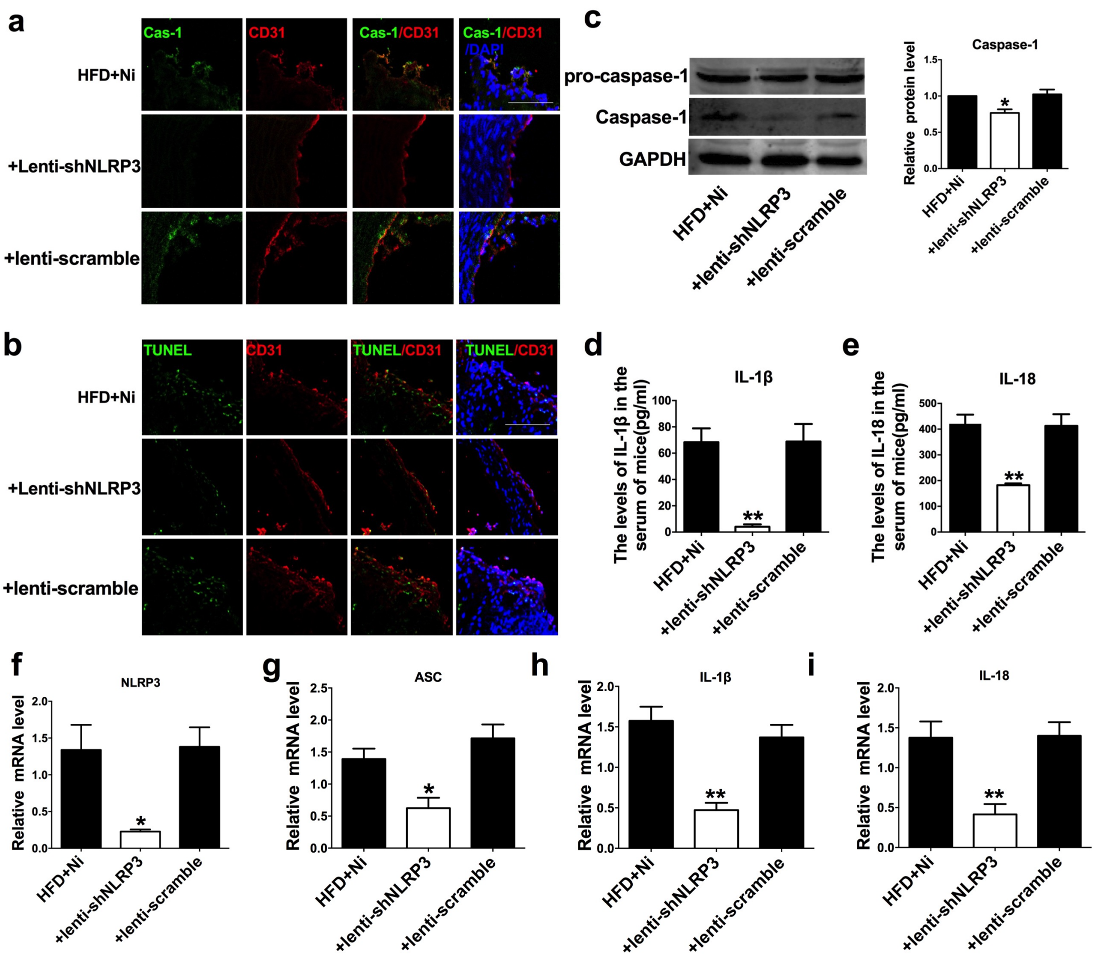

Figure S3. NLRP3 knockdown inhibits pyroptotic response of endothelial cells in the mice administrated with HFD and nicotine. (**a**) Double staining of Caspase-1 (green) co-located with CD31 (red) in atherosclerotic lesions of ApoE$^{-/-}$ mice induced with HFD+Ni and injected with NLRP3 shRNA or not. Magnification: 200×. (**b**) Double staining of CD31 (red) co-located with TUNEL (green) reaction. The nuclei were stained blue with DAPI. CD31was used as an endothelial marker. Scale bar＝100 μm. Magnification: 200×. (c) Western blot analysis of Pro-caspase-1 and Caspase-1 in different group. n=6 mice in each group. $*P < 0.\ 05$ vs HFD+Ni. (d-e) The serum concentration of IL-1β and IL-18 were determined by ELISA assay. Data are shown as mean ± SEM. n=6 mice in each group. $**P < 0.01$ vs HFD+Ni. (f-i) Pyroptosis-associated genes (NLRP3, ASC, IL-1β and IL-18) expression in intimal RNA of ApoE^{-}

/- mice in different groups. n=6 mice in each group. The data are presented as the mean ± SEM., *P<0.05. **P<0.01 vs HFD+Ni.

Supplementary Figure 4

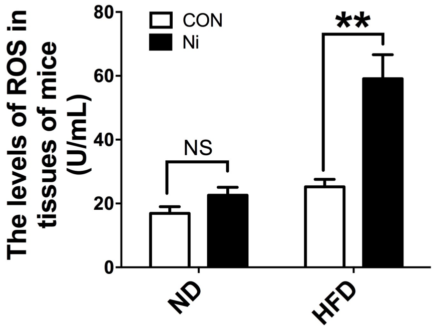

Figure S4. Detection of ROS levels in ApoE$^{-/-}$ mice treated with different conditions. **P< 0.01 vs control.

Supplementary Figure 5

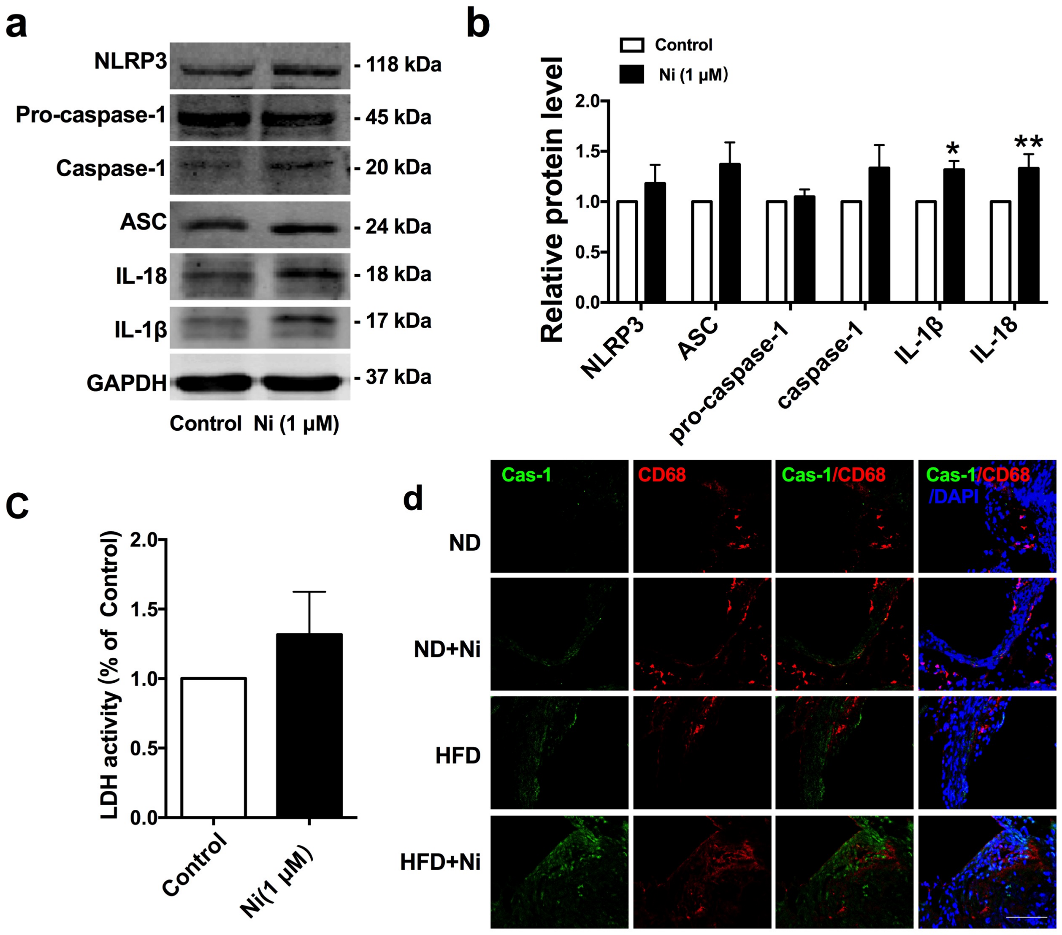

Figure S5. Pyroptosis-related proteins expression and LHD release in nicotine-

treated mouse macrophages (RAW264.7). (a) Representative western blotting bands for NLRP3, Pro-caspase-1, caspase-1, ASC, IL-18 and IL-1β in RAW264.7 cells treated with or without nicotine (1 μM) for 24 h. (**b**) Quantitative analysis of relative levels of pyroptosis-related protein expression. GAPDH was used as an internal control. (e) Pyroptotic cell was determined by LDH release in RAW264.7 cells treated with or without nicotine (1 μM) for 24 h. *P< 0.05. **P< 0.01 vs control. (d) Double staining of Caspase-1 (green) and CD68 (red) in atherosclerotic lesions of ApoE$^{-/-}$ mice in different groups. Scale bar＝100 μm. Magnification: 200×.

Supplementary Figure 6

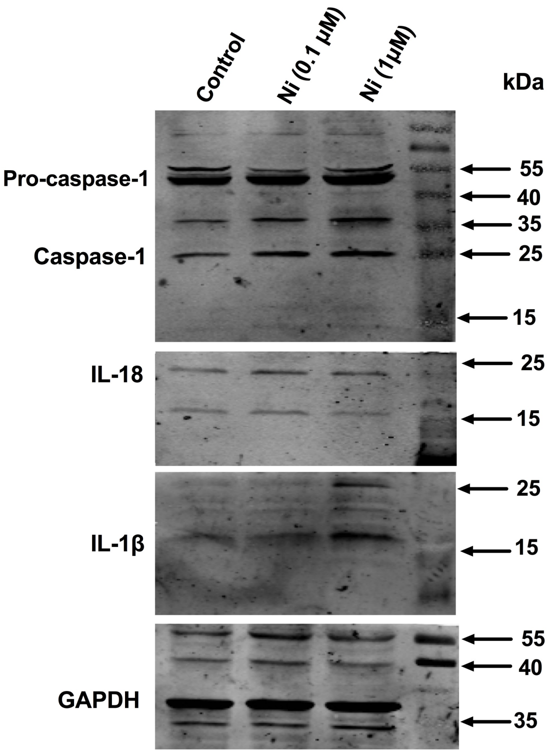

Figure S6. Full-length blots of Figure 3(a) in the main text. Effect of nicotine on the protein levels of Pro-caspase-1, Caspase-1, IL-18 and IL-1β in HAECs. Ni indicates nicotine.

Supplementary Figure 7

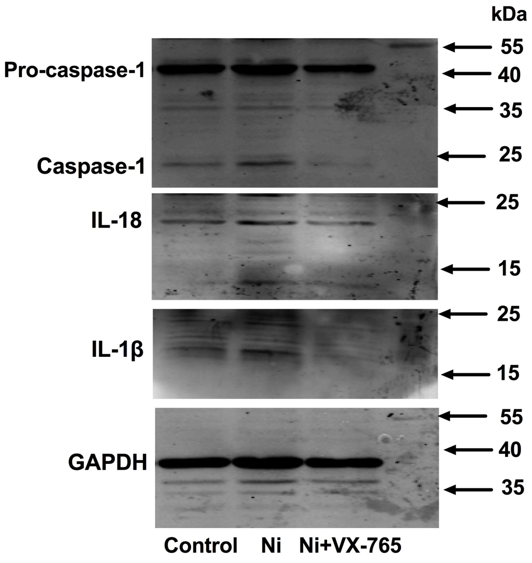

Figure S7. Full-length blots of Figure 4(a) in the main text. Caspase-1 inhibitor inhibited the activation of caspase-1 and maturation of IL-18 and IL-1β. Ni indicates nicotine.

Supplementary Figure 8

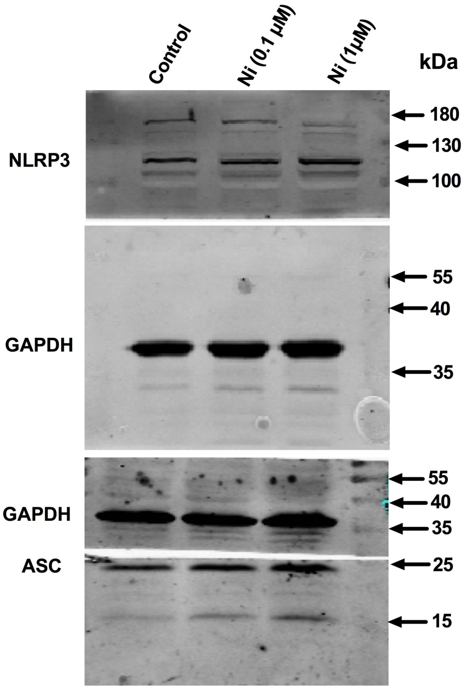

Figure S8. Full-length blots of Figure 5(a) in the main text. Effect of nicotine on the protein levels of NLRP3 and ASC in HAECs. Ni indicates nicotine.

Supplementary Figure 9

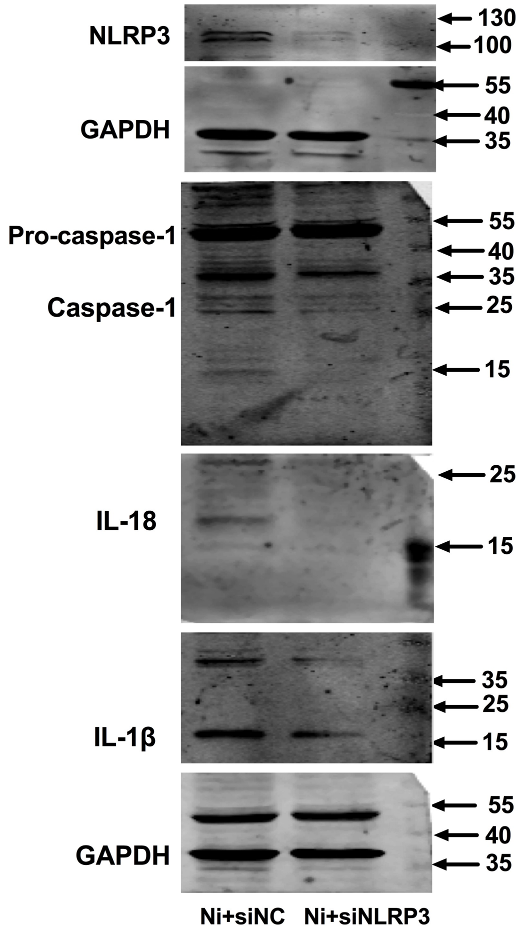

Figure S9. Full-length blots of Figure 5(c, e) in the main text. Effect of siNLRP3 on the protein levels of Pro-caspase-1, Caspase-1, IL-18 and IL-1β in HAECs. Ni indicates nicotine.

Supplementary Figure 10

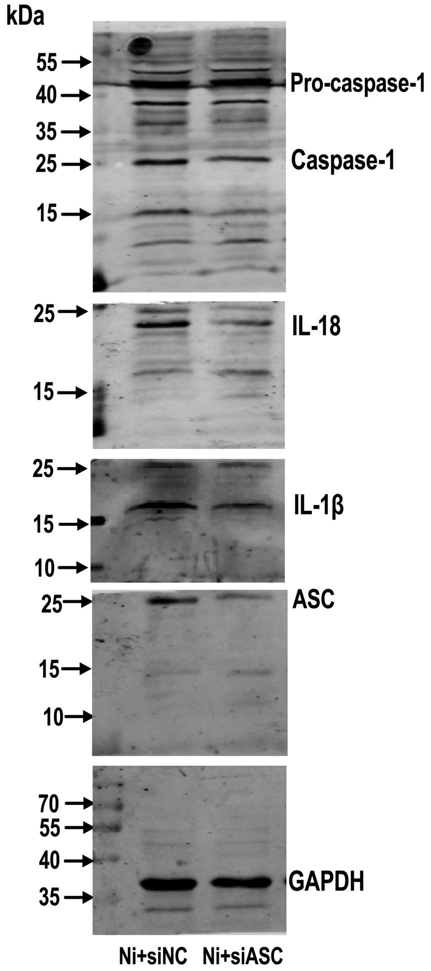

Figure S10. Full-length blots of Figure 6(a-b) in the main text. Effect of siASC on the protein levels of Pro-caspase-1, Caspase-1, ASC, IL-18 and IL-1β in HAECs. Ni indicates nicotine.

Supplementary Figure 11

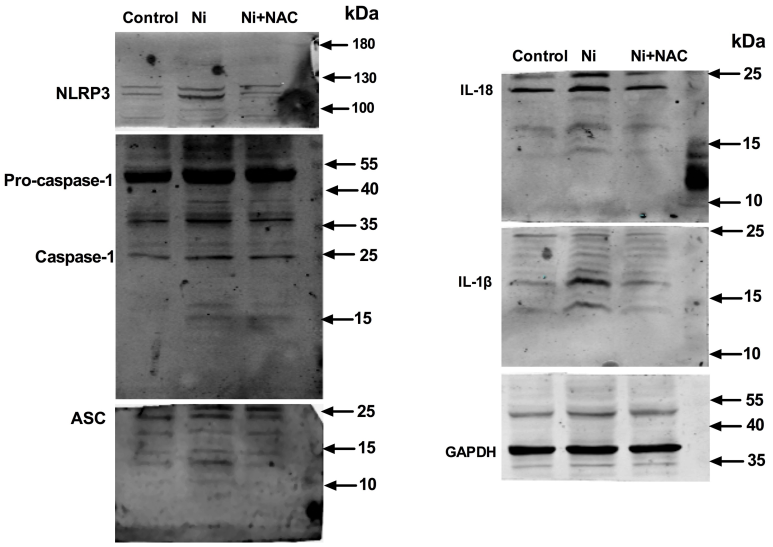

Figure S11. Full-length blots of Figure 7(c) in the main text. Effect of NAC on the protein levels of NLRP3, Pro-caspase-1, Caspase-1, ASC, IL-18 and IL-1β in HAECs. Ni indicates nicotine.
